# Application of Mobile Health Technologies to Prevent Unintentional Childhood Injuries: Scoping Review

**DOI:** 10.2196/94038

**Published:** 2026-08-14

**Authors:** Huan Wang, Fengjiao Pu, Dongjun Jing, Hangcheng Liu, Miao Li, Xixi Li

**Affiliations:** 1 Department of Nursing Mianyang Central Hospital, School of Medicine, University of Electronic Science and Technology of China Mianyang, Sichuan China; 2 Department of Obstetrics and Gynecology Mianyang Central Hospital, School of Medicine, University of Electronic Science and Technology of China Mianyang, Sichuan China; 3 School of Nursing North Sichuan Medical College Nanchong, Sichuan China

**Keywords:** child, mobile health, scoping review, technologies, unintentional injuries

## Abstract

**Background:**

Unintentional childhood injuries represent a major public health issue affecting the lives and health of children worldwide, with the risk and injury type dynamically changing with age and developmental stage. To effectively prevent unintentional injuries, intervention measures must be adjusted based on actual circumstances and kept up to date in real time. Mobile health (mHealth) technologies can meet this need, but their specific functions and intervention methods remain unclear at present.

**Objective:**

This study aimed to conduct a scoping review of research on the application of mHealth technologies in the prevention and emergency management of unintentional injuries among children, with a focus on identifying the types of mHealth technologies used, intervention contents, and outcome measures, thereby providing a reference for related studies.

**Methods:**

We systematically searched 5 databases—PubMed, Web of Science, Embase, Cochrane Library, and CINAHL—from their inception to December 31, 2025. Data extraction and synthesis were performed on the included studies.

**Results:**

Of 1085 articles, 15 (1.3%) met the inclusion criteria, and 1 additional reference was added during full-text review. Thus, 16 studies were included. The included studies comprised 9 (56.2%) randomized controlled trials, 2 (12.5%) quasi-experimental studies, 3 (18.8%) mixed methods studies, 1 (6.2%) qualitative study, and 1 (6.2%) descriptive study. mHealth technologies included apps, WeChat, mobile-based e-learning programs, and virtual reality combined with wearable devices. Their functions included health education, interactive communication, monitoring and reminders, behavioral training, questionnaires and record-keeping, and emergency response. Outcome measures included the incidence of unintentional injuries among children; knowledge, attitudes, and behaviors regarding injuries; other psychological and social indicators; and usability of mHealth technology.

**Conclusions:**

mHealth technologies have shown some progress in reducing unintentional injuries among children. Content based on this technology has demonstrated positive effects in reducing injury incidence, enhancing caregivers’ awareness, and improving related attitudes and behavioral capabilities. However, only 2 mobile apps are currently available to the public. Future research should optimize the functional design of mHealth, strengthen sustainability, explore long-term implementation methods, and conduct large-scale, long-term studies to further evaluate its impact on the incidence of unintentional injuries among children.

**Trial Registration:**

Open Science Framework 8QPR6; https://osf.io/8qpr6

## Introduction

### Background

Unintentional injuries are bodily harm caused by sudden events, including accidental falls, drowning, burns, suffocation, and poisoning [1]. It is estimated that unintentional injuries are the leading cause of death among children aged 1 to 19 years globally and the fifth leading cause of death among infants aged <1 year [2,3]. The 2019 Global Burden of Disease Study reveals that 205,000 children and adolescents aged 5 to 24 years died from unintentional injuries, with children aged 5 to 9 years facing the highest risk of death. According to China’s Under-5 Child Mortality Surveillance System reports, between 2010 and 2020, a total of 7925 children died from unintentional injuries, and the proportion of deaths among injuries rose from 15.2% in 2010 to 23.8% in 2020 [4]. It not only poses a severe threat to children’s lives and health but also imposes heavy economic burdens and psychological stress on families and society. The occurrence of unintentional injuries involves multiple factors, including the child’s age and developmental stage, the guardians’ educational and supervisory levels, and the safety conditions of the surrounding environment [5-7]. Among all the risk factors, age and developmental stage are important predictors, indicating that injury types and risk factors vary dynamically with the child’s age and developmental stage. For example, young children are more prone to unintentional injuries, and infants aged <1 year are commonly more prone to choking or suffocation; while children over 1 year of age are more likely to experience accidental falls or burns [6]. It is this characteristic that requires interventions for unintentional injuries to be timely adjusted and updated to the child’s age and developmental stage.

The World Health Organization published the World Report on Child Injury Prevention (2008), which outlines key interventions across multiple areas, including legislation, product safety, environmental modification, education, and first aid [8]. However, the strategies, both domestically and internationally, primarily rely on offline approaches that have limited coverage, target smaller populations, and yield short-term effects, wasting time and money and lacking sustainability. With the rapid development of IT, mobile health (mHealth) offers a new perspective and platform for preventing and controlling unintentional childhood injuries. mHealth refers to the use of smartphones, wearable devices, mobile apps, and other technologies to deliver health promotion, disease prevention, and public health management services to the public [9]. This technology offers convenience, real-time access, personalization, and intelligence, enabling dynamic updates to intervention content and long-term tracking, thereby addressing the shortcomings of traditional approaches and aligning with the requirements of unintentional injuries [10]. Currently, mHealth technologies are widely used in areas such as chronic disease management, rehabilitation, cancer care, and perinatal care, and show great promise [11-14].

In the field of child injury prevention, some studies have also attempted to develop mHealth interventions to prevent unintentional injuries in children. For example, Feng et al [15] used WeChat to regularly send information on the prevention of unintentional injuries and emergency strategies to parents. Cooray et al [16] used a mobile app to help parents of children aged <1 year improve their ability to prevent infant falls. Wong et al [17] used serious games to help children learn traffic safety signs and understand potential safety hazards in different areas. Because researchers use different mHealth technologies, the target populations, functional focuses, and intervention outcomes vary. A review is needed to clarify the format, advantages, effectiveness, and potential limitations of each technology. Currently, there are few systematic reviews in this field. Schulze et al [18] conducted a review of mobile apps for unintentional injuries in children aged <7 years, detailing the user groups, specific needs, and evaluation metrics of these apps. However, this study included only 5 papers published before 2021, resulting in limited evidence that does not comprehensively reflect the full scope of the field. Shetty et al [19] reviewed studies on interventions for unintentional injuries in children from 2013 to 2023, including 9 eHealth-based studies; however, they similarly failed to provide detailed descriptions of the technologies’ features. Overall, there remains a lack of systematic reviews that comprehensively describe the current status of mHealth technology applications, the types of technologies, and the effectiveness of interventions for preventing unintentional injuries among children.

### Objectives

This study aims to provide a systematic review of the current status of mHealth technology applications in interventions for unintentional injuries in children. The primary objectives are to (1) summarize the types of mHealth technologies used for unintentional injuries in children through a systematic review of the existing literature and (2) examine how these technologies are used in interventions for unintentional injuries in children, what the outcome measures of these interventions are, and how effective these interventions are.

## Methods

The study followed the Joanna Briggs Institute Manual for Scoping Reviews Framework and the PRISMA-ScR (Preferred Reporting Items for Systematic Reviews and Meta-Analyses extension for Scoping Reviews; Multimedia Appendix 1) [20]. The protocol was registered in Open Science Framework.

### Defining Research Questions

The review was guided by the following research questions:

1. What mHealth technologies are used in interventions for unintentional injuries among children? Which technologies are currently available?
2. What are the target populations and intervention content of studies on mHealth technology-based interventions?
3. What are the outcome measures for mHealth technologies?
4. How effective are mHealth technologies in preventing unintentional injuries among children?

### Definition of mHealth Technology

The concept of mHealth originates from eHealth, which refers to the use of information and communication technology to support health care services and information dissemination [21]. With the widespread adoption of smartphones and tablets, eHealth services—which previously relied on fixed devices or locations—are gradually shifting toward mobile devices as their primary platform. This study follows the World Health Organization’s definition, which defines mHealth as health care and public health practices supported by mobile devices [9]. Whether a technology falls under the category of mHealth is primarily determined by whether it is accessed via mobile devices such as smartphones and tablets. Accordingly, in addition to common apps and wearable devices, this study includes within the scope of mHealth any technology accessed or operated through mobile portable devices, such as internet platforms, serious games, and virtual reality (VR) technology.

### Search Strategy

A systematic search was conducted across 5 databases: PubMed, Web of Science, Embase, Cochrane Library, and CINAHL. A combination of subject headings and free-text terms was used, with additional reference tracing. The search covers each database from its inception to December 31, 2025. Search terms included “children,” “child,” “infant,” “child, preschool,” “accidental injuries,” “unintentional injuries,” “accidental,” “injuries,” “mobile applications,” “app,” “mobile phone,” “social media,” “digital health,” “digital media,” “telemedicine,” “internet,” “text messaging,” “virtual,” “digital technology,” “artificial intelligence,” “Web,” “WeChat,” “online social network,” “message,” and “smartphone.” The search terms and strategy used are presented in Multimedia Appendix 2.

### Literature Inclusion and Exclusion Criteria

Literature inclusion criteria were established based on population, concept, and context principles: (1) the study population comprised children, child caregivers, or child educators; (2) the study concept involved mHealth technology research; (3) the context pertained to studies related to unintentional injuries among children. The study types included original research such as randomized controlled trials, quasi-experimental studies, mixed method studies, and observational studies. Exclusion criteria were as follows: (1) duplicate publications; (2) if there are multiple papers reporting on the same study, only the one reporting the final results will be included; (3) study protocols; (4) publications for which the full text is unavailable; and (5) publications not in Chinese or English.

### Literature Screening and Data Extraction

Literature screening was conducted using NoteExpress software (version 4.1.0.10030; Beijing Aegean Software), with 2 researchers independently performing initial and duplicate screening. Disagreements were resolved through discussion with a third researcher. Extracted data included authors, publication year, country, study type, study subjects, sample size, app type, study duration, specific implementation methods, control group measures, and outcome measures.

## Results

### Literature Search Results

**Figure 1:**
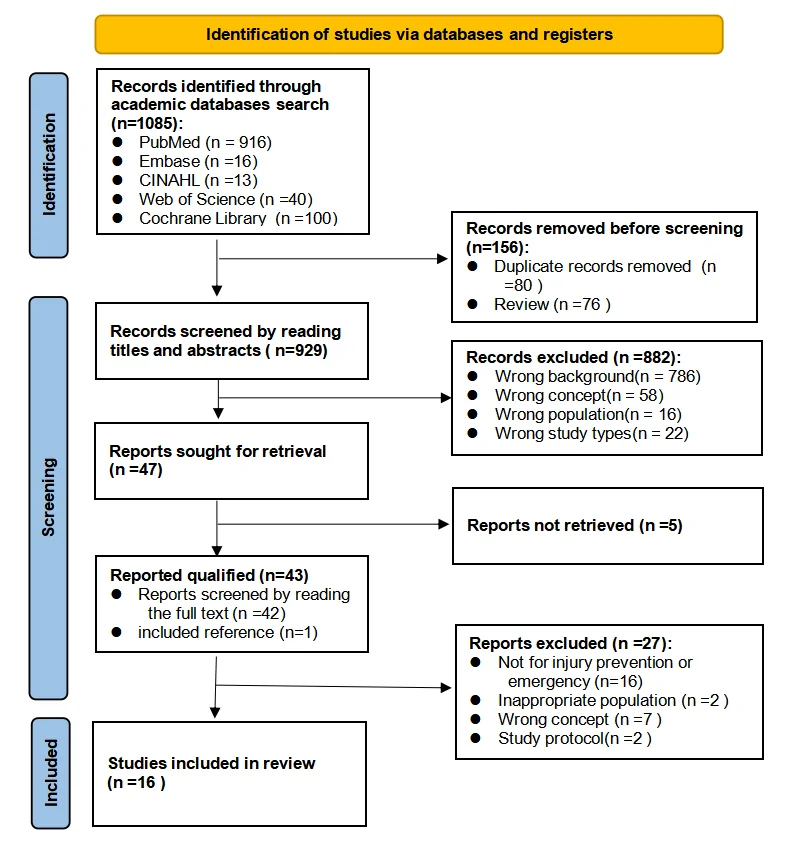
PRISMA (Preferred Reporting Items for Systematic Reviews and Meta-Analysis) flow diagram.

The initial search yielded 1085 articles. After removing duplicates and reviews, 929 articles remained. Following a review of titles and abstracts, 882 articles were excluded. After reviewing the full texts, 25 articles were excluded for irrelevant research topics, 2 were excluded as protocols, and 5 could not be retrieved. There were 15 remaining articles, and 1 article was included through reference tracing. Finally, a total of 16 articles were ultimately included. The literature screening process is illustrated in Figure 1.

### Basic Characteristics of Included Literature

All included studies were in English. The collection comprised 9 randomized controlled trials [15,17,22-28], 2 quasi-experimental studies [29,30], 3 mixed method studies [16,31,32], 1 qualitative study [33], and 1 descriptive study [34]. Geographically, 6 studies were from China [15,17,25,26,28,30], 3 from the United States [23,24,32], 1 from South Korea [22,29,34], 2 from Australia [16,27], and 1 each from the United Kingdom [33] and Germany [31]. Specific characteristics are shown in Multimedia Appendix 3.

### Types of mHealth Technologies

#### Mobile Apps

Twelve studies [16,17,23-25,27-29,31-34] used apps as their delivery platform. Among these, 6 apps [16,17,23,25,27,28,33,34] were specifically developed by the research teams to meet their own research needs. Ning et al [25] and He et al [28] used the same app (Bao Hu San, Protective Umbrella), which was developed by Ning et al [25] for injury prevention education among urban child caregivers; He et al [28] adapted and improved it based on that study to address rural conditions. The game app Safe City, developed by Wong et al [17], is designed to help children develop proper traffic safety behaviors. This app is currently available for download via a web browser, but users must provide a mobile phone number to register. The apps developed by Kang et al [34] and Jones et al [33] provide education on the prevention of unintentional injuries to schoolteachers and mothers, respectively; however, all of them are currently in the development and usability testing phases. A total of 4 apps [24,27,29,31,32] have been developed by relevant organizations in advance. The app used by McKenzie et al [24] and Roberts et al [32] (Make Safe Happen) was developed by the US Centers for Injury Prevention and Control and is specifically designed for parents of children aged 0 to 12 years. Its features include age-appropriate safety information, room safety checklists, links to purchase safety products, the ability to create shopping lists and set calendar reminders, and the option to add a poison control hotline to phone contacts with a single tap. However, this app is no longer being updated and is unavailable for download. The app used in the study by Kim and Cho [29] was developed by the South Korean Ministry of Education to help children handle emergencies they might encounter at school or in daily life; the download link was provided in the literature but is currently unavailable. Müller et al [31] conducted an evaluation of the Vergiftungsunfalle bei Kindern (“child poisoning accidents”) app, developed by the German Federal Institute for Risk Assessment in 2013. This app is designed for caregivers and primarily provides information on toxic substances, prevention advice, and emergency call functions. This app is currently available for download on the Google Play Store and the App Store. The Cool Runnings app, used in the study by Burgess et al [27], was developed by a research team at the University of Queensland’s Center for Pediatric Burns and Trauma to raise mothers’ awareness of scald injuries from hot beverages and improve their emergency response capabilities; however, the app is no longer being updated and is unavailable for download.

#### WeChat

Two studies [15,26] used WeChat to provide relevant resources for child caregivers. Feng et al [15] established WeChat groups and an official account to provide caregivers with resources on injury prevention; the WeChat official account registered for this study is currently searchable but is no longer being updated. In the other study, Yan et al [26] provided on-site training to child caregivers at schools and subsequently used WeChat groups to regularly distribute learning materials on child restraint systems.

#### Mobile-Based e-Learning Program

Choi and Ahn [22] developed an e-learning program using Storyline 360 e-learning software and generated a course link. The course includes videos, audio, and slides, and parents can access the link via computers, tablets, and smartphones to study at their convenience. In addition, the course supports repeat viewing and progress tracking, allowing caregivers to review the material whenever needed. This program is not available to the public.

Mobile-based VR technology and wearable devices: Schwebel et al [30] developed a VR pedestrian safety training system. This system uses a smartphone as both the platform for running VR content and the display device; the smartphone is inserted into a Viewmaster VR stereoscopic display equipped with goggles and a joystick to run a virtual crosswalk environment. Children undergo 12 days of repeated crossing training in the classroom. The system provides immediate feedback based on their crossing performance and automatically records behavioral data such as collisions and unsafe crossings. This technology is currently still in the experimental stage and has not yet been made available to the public.

### Study Duration and Follow-Up Periods

The duration of the included studies varied. Specifically, for studies assessing mHealth interventions, the intervention periods ranged from 1 week to 12 months. In contrast, among studies adopting observational or cross-sectional designs, only 2 reported the study duration: 7-10 days [32] and 3 months [16], respectively.

### Target Population

Eleven studies [15,16,22,24-28,31-33] focused on caregivers of children, covering those caring for children aged 0 to 12 years; 4 studies [17,23,29,30] targeted children directly, with participants ranging in age from 5 to 13 years; and 1 study [34] was conducted with schoolteachers.

### Injury Type Addressed

Nine studies [15,22,24,25,28,29,32-34] addressed unintentional injuries as a whole, including common types such as choking, aspiration, falls, poisoning, drowning, and burns. The remaining 7 studies focused on specific types of injuries, including 2 on traffic accidents [17,26], 1 on burns and scalds [27], 1 on accidental falls among infants [16], 1 on accidental poisoning [31], and 1 on the prevention of bicycle injuries and animal bites [23].

### Theoretical Framework

The included studies drew upon behavior change theory [16,23], Orem’s self-care theory [29], and the Haddon model [15] to guide the design of learning content; Choi and Ahn [22] designed their learning program based on the analysis, design, development, implementation, and evaluation (ADDIE) model; Wong et al [17] developed a game app by integrating social cognitive theory with an experiential game model; Ning et al [25] designed an app by combining behavior change theory, the Haddon model, and the rational analysis framework for mobile education; Müller et al [31] evaluated the app’s perceived usefulness, perceived ease of use, trust, and intention to use based on the technology acceptance model.

### Intervention Content

#### Health Education

A total of 15 studies [15-17,22-29,31-34] used mHealth technologies to deliver health education on unintentional injuries to children and caregivers via text, images, audio, video, and games. The interventions included raising awareness of injuries, changing attitudes or beliefs regarding injury supervision, providing home environment checklists and hazard-identification tools, disseminating knowledge and skills on prevention, and teaching emergency response methods following an incident. The curriculum designed by Choi and Ahn [22] covered 6 scenarios: the bedroom, living room, bathroom, kitchen, outdoors, and inside a vehicle. Each scenario included prevention knowledge for injury types such as choking, aspiration, falls, poisoning, physical injuries, drowning, and burns. The program also provided information on safety products but did not include purchase links. Ning et al [25] and He et al [28] used the same app to provide education on unintentional injuries to caregivers of urban and rural children, respectively. The Make Safe Happen program used by McKenzie et al [24] and Roberts et al [32] categorizes children by age into 5 groups: 0 to 11 months, 12 to 23 months, 2 to 4 years, 5 to 9 years, and 10 to 12 years. It develops personalized safety information tailored to the types of injuries most common in each age group and provides home environment checklists to help caregivers identify potential hazards. Feng et al [15] conducted a study that used WeChat groups and official accounts to regularly send weekly updates to parents regarding the prevention of common childhood accidents, aiming to enhance their awareness of such injuries. Wong et al [17], Burgess et al [27], and Dixon et al [23] incorporated game-based simulations of relevant scenarios to improve caregivers’ or children’s awareness of and ability to prevent unintentional injuries. Furthermore, Cooray et al [16] focused on the differences in fall risks between infants and children over 1 year of age. They provided caregivers with customized safety information centered on four themes—safe feeding, furniture use, infant product safety, and the environment—demonstrating a personalized educational strategy tailored to population characteristics, which further highlights the refinement and personalization of health education.

#### Interactive Communication

Five studies [15,16,23,25,28] provided communication support channels via mHealth technology for caregivers of children. Through these platforms, caregivers can raise questions encountered while implementing accident prevention measures in user forums, discussion areas, or expert consultation channels, and receive insights from experienced caregivers or targeted guidance and answers from health care professionals and researchers. Ning et al [25] focused on facilitating mutual communication among users and between users and researchers. Building on this, He et al [28] optimized expert consultation and customer service functions, added online forums and themed activities, and strengthened interactions among researchers, experts, and caregivers. Feng et al [15] collected caregivers’ questions via WeChat groups, with community doctors providing responses within 48 hours to ensure timely guidance. Cooray et al [16] focused on collaborative communication, with researchers and caregivers jointly developing emergency response plans to enable rapid and effective action in the event of an infant fall. Dixon et al [23] encouraged increased communication between children and their caregivers regarding the prevention of bicycle injuries and pet bites.

#### Behavioral Training

Five studies [17,23,26,27,30] used mHealth technologies—such as WeChat, smartphone-based VR systems, and gaming apps—to deliver injury prevention training to children and caregivers. The content included safe road-crossing skills for children, proper child safety seat installation methods for caregivers, and the identification and management of household safety hazards. Schwebel et al [30] used a smartphone-based VR system to train children’s road-crossing behavior, enabling their performance to meet adult standards. Wong et al [17] used a game to simulate road-crossing scenarios and train children’s safety behaviors; to prevent game addiction, the study implemented an energy-point mechanism that required a 2-hour wait before restarting once energy was depleted. Dixon et al [23] combined an app with a game to simulate street environments, indoor and outdoor residential settings, animal shelters, and parks, requiring children to learn and practice safe cycling skills and how to interact with animals while responding to various risk scenarios. Burgess et al [27] used gamification techniques to help caregivers master measures to prevent accidental poisoning and burns in children.

#### Monitoring and Reminders

Six studies [16,24,27,29,32,34] used mobile apps or WeChat to monitor and provide reminders about the safety behaviors or learning progress of children and their caregivers. McKenzie et al [24] and Roberts et al [32] guided caregivers to set up reminders in the app for testing and replacing safety equipment and monitored their implementation. Kim and Cho [29] established a classroom log system within the app, requiring students to record their completion of the previous day’s learning tasks daily. Cooray et al [16] used task tracking lists and progress dashboards to show parents the completion status of safety tasks and sent regular SMS text message reminders prompting users to read relevant articles. Burgess et al [27] required caregivers to upload photos of home equipment between push notifications to assess the safety status of the equipment, and to complete pop-up quizzes to reinforce injury prevention knowledge. Kang et al [34] designed a child safety checklist for teachers to use for self-assessment of safety behaviors, with the system providing feedback through colors or markers.

#### Other Intervention Components

In terms of data collection and user settings, 2 studies [25,28] used mobile platforms to collect data on childhood accidents, survey responses, and user feedback online. Among these, Bao Hu San [25,28] also allowed users to customize the app interface to suit their personal preferences, thereby enhancing user satisfaction. Regarding emergency features, Make Safe Happen [24,32] included a poison control hotline that could be added to a user’s phone contacts with a single tap; Jones et al [33] provided emergency phone numbers for caregivers to look up; and Müller et al [31] included a 1-tap button to call a poison control center and provided guidelines for pediatric care to help caregivers seek immediate medical assistance.

### Outcome Measures and App Effectiveness

#### Overview

The studies included in this review used various methods to evaluate the effectiveness of mHealth technology interventions, including researcher-designed questionnaires, participant self-reports, and individual or focus group interviews. Consequently, there were significant variations in the naming and measurement of outcome measures. For ease of presentation, this study categorized these measures into 5 groups.

Incidence of unintentional injuries among children

Three studies [15,25,28] measured the incidence of unintentional injuries in children as the primary outcome, using caregivers’ self-reported data for statistical analysis. Among these, He et al [28] and Feng et al [15] both found that the incidence of unintentional injuries in the intervention group was lower than that in the control group after the intervention was completed; however, the incidence of falls in the intervention group led by He et al [28] was slightly higher than that in the control group (adjusted risk ratio 1.17, 95% CI 1.06-1.30); whereas in the 6-month follow-up period reported by Ning et al [25], although the incidence in the intervention group decreased from 8.76% to 8.11%, the difference was not statistically significant (*P*=.59).

#### Knowledge, Attitudes, and Behaviors

##### Knowledge Level

Eight studies [15,17,22-24,26,27,29] measured knowledge level as one of the outcome indicators, primarily including awareness of unintentional injuries [22], knowledge of prevention and safety [17,23,24], knowledge of child restraint system use [26], and first aid knowledge in the event of an accident [15,27,29]. All were assessed using questionnaires designed by the researchers. Among these, the studies by Choi and Ahn [22], Feng et al [15], and Wong et al [17] did not observe any significant statistical differences.

##### Attitudes

Three studies [25,26,28] used attitude levels as outcome measures, including caregivers’ attitudes toward accident prevention [25,28] and the use of child restraint systems [26]. Among these 3 studies, only the study by Ning et al [25] did not show statistically significant differences.

##### Behavior

Eleven studies [15-17,22-26,28,30,32] used behavioral competence of children and their caregivers as one of the outcome measures, including injury prevention and safety behaviors [17,22-25,32], caregiver supervision behaviors [15,28], traffic safety behaviors [30], use of child restraint systems [26], and behaviors with the potential to reduce the risk of infant falls [16]. Regarding the measurement of this outcome indicator, with the exception of Wong et al [17], who observed no significant differences, all other studies reported statistically significant differences.

#### Psychosocial Outcomes

Wong et al [17] used the Strengths and Difficulties Questionnaire to assess psychosocial problems in children. While the study did not observe statistically significant changes in these outcomes following the intervention, an analysis of the dose-response relationship revealed that cumulative game scores were associated with improvements in internalizing problems at the 3-month mark of the intervention, suggesting that serious games can enhance children’s psychosocial functioning. Feng et al [15] assessed improvements in caregivers’ beliefs regarding the prevention of unintentional injuries following the intervention. This indicator was divided into 3 components based on relevant literature: injury attribution beliefs, preventability beliefs, and responsibility beliefs. The results showed that mHealth technology can improve caregivers’ beliefs regarding the preventability of unintentional injuries (*β*=.344, 95% CI 0.152-0.537; *P*<.001). Schwebel et al [30] measured children’s self-efficacy when crossing the street using both questionnaires and behavioral observations. The study showed that the proportion of children able to safely cross busy streets increased from 3.6% to 19.6% (odds ratio [OR] 4.7; *P*=.002).

#### Other Indicators

Other indicators included home environment safety (*β*=3.38, 95% CI 2.51-4.16; *P*<.001) [28], the use rate of child restraint systems (OR 0.29, 95% CI 0.11-0.79; *P*=.016) [26], and caregivers’ awareness of injury prevention (which increased significantly from 63% before the intervention to 81%, *P*<.01) [32]. Additionally, 2 studies [16,32] identified barriers and facilitators to caregivers’ adoption of unintentional injury prevention measures through focus group interviews. Both studies indicated that simplifying tasks and increasing app reminders and feedback features could promote the implementation of injury prevention measures; conversely, incomplete functional content, impractical recommendations, time constraints, high costs, cumbersome tasks, and a lack of family support hindered their implementation.

#### Usability of mHealth Technologies

Eleven studies [15-17,22-26,28,30,32] reported metrics related to mHealth technologies, primarily including user satisfaction [22,26,34], user engagement [16,23,27,32,33], user experience [30-33], user acceptance [16,23], the educational effectiveness of smartphones (frequency, practicality, interest, and ease of understanding) [29], economic losses resulting from childhood accidents [25], and the economic costs of the apps [15,25], as well as the identification of facilitators and barriers affecting user acceptance through targeted individual interviews [31]. The specific results are presented in Multimedia Appendix 3.

## Discussion

### Overview of mHealth Technologies for Unintentional Childhood Injuries

The results of this study indicate that mHealth technologies currently used in the field of unintentional childhood injuries include mobile apps, WeChat, mobile-based e-learning programs, VR technology, and wearable devices. Apps and WeChat are primarily targeted at child caregivers, as their busy daily schedules make it difficult to set aside time for in-person training. Apps and WeChat platforms offer convenience and quick access to health information, helping caregivers learn in spare moments or obtain necessary information promptly when needed. Among the mobile apps included in this study, with the exception of the 3 apps cited by Wong et al [17], Dixon et al [23], and Kim and Cho [29], all others were designed for child caregivers, with one specifically targeting teachers. In terms of functional design, health education or regular learning content is primarily delivered through audio, video, text, images, and gamified formats; user forums or comment sections facilitate interaction between users and researchers or health care professionals; check-in features and progress bars are used to monitor users’ learning progress and adherence to safety measures; additionally, some apps include emergency contact information or links to purchase safety products. The results of this study indicate that participants demonstrated high levels of engagement, acceptance, satisfaction, and positive user experience with the apps they used. For children, games and VR technology are currently widely adopted. This study included 2 gaming apps, one VR app combined with wearable devices, and one health education-focused app. Key features included simulations of real-world environments such as streets, residential areas, dog shelters, and parks, designed to help children develop appropriate safety behaviors and prevent traffic accidents and pet bites. The health education-focused app provides children with emergency response knowledge for various situations, covering 8 categories of emergency scenarios: school safety, off-campus safety, responding to violent incidents, traffic accidents, accidental poisoning, basic first aid skills, natural disasters, and evacuation drills—totaling 48 specific emergencies. Each scenario includes a brief description and response recommendations, with some supplemented by instructional videos. Compared with traditional lecture-style education, children demonstrate greater acceptance of this engaging, interactive learning method.

### Positive Effects and Development Requirements

This study describes the effectiveness of mHealth-based interventions in preventing unintentional injuries among children. The results indicate that such interventions have a positive impact on reducing the incidence of unintentional injuries among children, enhancing the safety knowledge of children and caregivers, and improving their safety attitudes and behaviors. However, among the mHealth technologies currently used for interventions targeting unintentional injuries in children, only the Vergiftungsunfälle bei Kindern and Safe City apps are available for download and use via app stores or web browsers. The remaining technologies are either not publicly released or have ceased to be updated and maintained. The development and long-term maintenance of mHealth apps require significant investments of time, effort, and funding, while the actual social and health benefits are relatively limited, making it difficult for many projects to sustain operations [35]. Furthermore, mHealth technologies can overcome spatial and temporal constraints and address the shortfall of caregivers who are unable to participate in in-person training due to time constraints through online interventions. The online intervention model itself may experience low user adherence, with some users potentially losing motivation to continue participating after initial use [36]. Although this study did not assess user adherence, based on the dropout rates reported by Feng et al (24.38%) [15], Ning et al (32.19%) [25], and Burgess et al (51%) [27], long-term user adherence may be relatively low, which could be one of the reasons why mHealth programs fail to operate sustainably over the long term. The World Health Organization advocates that countries incorporate mHealth into their national public health strategies [37]. The “Action Plan for Injury Prevention and Control (2026-2030)” issued by China’s National Center for Disease Control and Prevention also emphasizes exploring and promoting appropriate technologies and strengthening education on child injury prevention and control skills, providing clear policy guidance for mHealth interventions. Therefore, it is recommended that future research leverage national public health programs to conduct long-term, large-scale studies on mHealth technologies. Relying on the public health system to overcome cost and adherence barriers can promote the sustainable application of mHealth interventions.

### Outcome Measures and Assessment Tools

Regarding the evaluation of the effectiveness of mHealth technologies, most studies have focused on caregivers’ knowledge, attitudes, and behaviors as primary outcome measures, while only 3 studies have primarily examined the incidence of unintentional injuries in children. The occurrence of unintentional injuries in children is influenced by multiple factors, including the home environment, the level of supervision, and the child’s developmental stage. To detect statistically significant differences, large sample sizes and long-term follow-up may be required, resulting in high research costs and significant implementation challenges [38,39]. Most of the studies included in this review had sample sizes ranging from 100 to 200 participants, with intervention durations of approximately 1 week to 3 months, which may not meet the basic requirements for conducting effective statistical tests for this outcome. Furthermore, regarding the selection of assessment tools, the studies included in this review all used self-developed or adapted questionnaires. Since the data rely on self-reports from caregivers, there is potential for bias and subjectivity, which may lead to a lack of comparability across study results [35,40]. Regarding the assessment of mHealth technology usability metrics, the literature included in this study generally reported favorable evaluations of these metrics by children and their caregivers across multiple dimensions, including user acceptance, engagement, user experience, and satisfaction. However, these evaluations were also largely based on assessment tools and criteria developed by the researchers themselves, lacking uniformity and comparability. Only the studies by Kang et al [34] and Müller et al [31] conducted standardized assessments of app usability and user satisfaction using the validated User Interaction Satisfaction Scale and the System Usability Scale, respectively. Currently, the academic community has developed a variety of assessment tools with good reliability and validity for the systematic evaluation of the usability of mHealth technologies. Therefore, it is recommended that future studies adopt these standardized, validated assessment questionnaires wherever possible to enhance the scientific rigor and comparability of research findings [41].

### Limitations

This study is a scoping review, so the quality of the included literature was not assessed. Additionally, the literature search was limited to English-language publications, which may have led to incomplete coverage of studies from certain regions or specific populations. Finally, this study focused on research that has been officially published and contains complete methodological information; gray literature, which often exists in the form of preliminary abstracts or unpublished data, usually does not meet the inclusion criteria for this review, so it was not systematically searched.

### Conclusions

This study provides a scoping review of the current use of mHealth technologies for the prevention and control of unintentional injuries among children. The findings indicate that mHealth technologies play a positive role in improving the knowledge, attitudes, and behaviors of children and their caregivers regarding unintentional injuries. However, only 2 mobile apps are currently available to the public. It is recommended that future efforts continue to optimize the functional design of mHealth, strengthen the technology’s sustainability, explore methods that can be implemented over the long term, and conduct large-scale, long-term studies to further evaluate the impact of mHealth on the incidence of unintentional injuries among children.

## Appendix

Multimedia Appendix 1 PRISMA-ScR checklist.

Multimedia Appendix 2 Search strategies.

Multimedia Appendix 3 Basic characteristics of included studies.

## Abbreviations

ADDIE: Analysis, Design, Development, Implementation, and Evaluation
mHealth: mobile health
OR: odds ratio
PRISMA-ScR: Preferred Reporting Items for Systematic Reviews and Meta-Analyses extension for Scoping Reviews
VR: virtual reality
