# Supplementary material for: Application of Mobile Health Technologies to Prevent Unintentional Childhood Injuries: Scoping Review
Source: JMIR Mhealth Uhealth. 2026 Aug 14;14:e94038. doi: 10.2196/94038 (PMC13525362; doi:10.2196/94038)
Supplement: Supplementary file 2 [file mhealth_v14i1e94038_app2.docx]

Multimedia Appendix 1. search strategies

| 1. PubMed | |
| --- | --- |
| #1 | (((((("Mobile Applications"[Mesh]) OR "Social Media"[Mesh]) OR "Digital Health"[Mesh]) OR "Digital Media"[Mesh]) OR "Telemedicine"[Mesh]) OR "Internet"[Mesh]) OR "Text Messaging"[Mesh] |
| #2 | ((((((((((("Mobile Phone"[Title/Abstract]) OR (APP[Title/Abstract])) OR (T-elemedicine[Title/Abstract])) OR (Virtual[Title/Abstract])) OR ("Digital T-echnology"[Title/Abstract])) OR ("artificial Intelligence"[Title/Abstract])) OR (Web[Title/Abstract])) OR (Wechat[Title/Abstract])) OR ("Online Soc-ial Network"[Title/Abstract])) OR (message[Title/Abstract])) OR ("social media"[Title/Abstract])) OR (smartphone[Title/Abstract]) |
| #3 | #1 OR #2 |
| #4 | "Accidental Injuries"[Mesh] |
| #5 | ((("Unintentional injuries"[Title/Abstract])) OR (Accidental[Title/Abstract])) OR (Injuries[Title/Abstract]) |
| #6 | #4 AND #5 |
| #7 | (("Child"[Mesh]) OR "Infant"[Mesh]) OR "Child, Preschool"[Mesh] |
| #8 | Children[Title/Abstract] |
| #9 | #7 AND #8 |
| #10 | #3 AND #6 AND #9 |

| 2. Web of Science | |
| --- | --- |
| #1 | TS=(Child OR Infant OR "Child, Preschool" OR Children) |
| #2 | TS=(Accidental Injuries OR "Unintentional injuries") |
| #3 | TS=(Mobile Applications OR "Social Media" OR "Digital Health" OR "Digital Media" OR Telemedicine OR Internet OR "Text Messaging" OR Mobile Phone OR APP OR Telemedicine or Virtual OR "Digital Technology" OR artificial Intelligence OR Web OR Wechat OR Online Social Network OR message) |
| #4 | #1 AND #2 AND #3 |

| 3. Embase | |
| --- | --- |
| #1 | child:ab,ti OR infant:ab,ti OR 'child, preschool':ab,ti OR children |
| #2 | accidental AND injuries:ab,ti OR 'unintentional injuries' |
| #3 | (mobile AND applications:ab,ti OR 'social media':ab,ti OR 'digital health':ab,ti OR 'digital media':ab,ti OR telemedicine:ab,ti OR internet:ab,ti OR 'text messaging':ab,ti OR mobile) AND phone:ab,ti OR 'app'/exp OR app |
| #4 | #1 AND #2 AND #3 |

| 4. Cochrane Library | |
| --- | --- |
| #1 | Child:ab,ti,kw OR Infant:ab,ti,kw OR "Child, Preschool":ab,ti,kw OR Children |
| #2 | Accidental Injuries:ab,ti,kw OR "Unintentional injuries" |
| #3 | Mobile Applications:ab,ti,kw OR "Social Media":ab,ti,kw OR "Digital Health":ab,ti,kw OR "Digital Media":ab,ti,kw OR Telemedicine:ab,ti,kw OR Internet:ab,ti,kw OR "Text Messaging":ab,ti,kw OR Mobile Phone:ab,ti,kw OR APP:ab,ti,kw OR Telemedicine:ab,ti,kw or Virtual:ab,ti,kw OR "Digital Technology":ab,ti,kw OR artificial Intelligence:ab,ti,kw OR Web:ab,ti,kw OR Wechat:ab,ti,kw OR Online Social Network:ab,ti,kw OR message |
| #4 | #1 AND #2 AND #3 |

| 5. CINAHL | |
| --- | --- |
| S1 | MH (MH "Child") OR MH (MH "Child, Preschool") OR MH (MH "Infant") OR TI ("Child" OR "Child, Preschool" OR "Infant" OR "Childern") OR AB ("Child" OR "Child, Preschool" OR "Infant" OR "Childern") |
| S2 | MH (MH "Accidental Injuries") OR TI ("Accidental Injuries" OR "Unintentional injuries") OR AB ("Accidental Injuries" OR "Unintentional injuries") |
| S3 | MH (MH "Mobile Applications") OR MH (MH "Social Media") OR MH (MH "Digital Media" OR MH "Digital Health") OR MH (MH "Telemedicine" OR MH "Telehealth") OR MH (MH "Internet") OR MH (MH "Internet") OR TI ("Mobile Applications" OR "Social Media" OR "Digital Media" OR "Digital Health" OR "Telemedicine" OR "Telehealth" OR "Internet" OR "Internet" OR "Mobile Phone" OR "APP" OR "Telemedicine" OR "Virtual" OR "Digital Technology" OR "artificial Intelligence" OR "Web" OR "Wechat" OR "Online Social Network" OR "message") OR AB ("Mobile Applications" OR "Social Media" OR "Digital Media" OR "Digital Health" OR "Telemedicine" OR "Telehealth" OR "Internet" OR "Internet" OR "Mobile Phone" OR "APP" OR "Telemedicine" OR "Virtual" OR "Digital Technology" OR "artificial Intelligence" OR "Web" OR "Wechat" OR "Online Social Network" OR "message") |
| S4 | S1 AND S2 AND S3 |
