# Supplementary material for: Application of Mobile Health Technologies to Prevent Unintentional Childhood Injuries: Scoping Review
Source: JMIR Mhealth Uhealth. 2026 Aug 14;14:e94038. doi: 10.2196/94038 (PMC13525362; doi:10.2196/94038)
Supplement: Supplementary file 3 [file mhealth_v14i1e94038_app3.docx]

Multimedia Appendix 3.The basic characteristics of the literature.

| Literature  Year | Country | Type | Population | Sample | Technology | Development Process | Intervention | Control | Duration | Outcome  Measure | Key Results |
| --- | --- | --- | --- | --- | --- | --- | --- | --- | --- | --- | --- |
| Kim and Cho [29]  2017 | South Korea | Quasi-experimental study | Grades 5-6 students | 44/42 | App | Developed by the South Korean Ministry of Education, the download link is provided in the text, but the link is currently unavailable. | ①⑥ | Traditional lecture-based education | 6 weeks | ^e^Knowledge  ^w^EES | The experimental group scored significantly higher than the control group in knowledge retention (*F=*7.96, *P*=.01). Smartphone-based education improved from “moderate” one week after the intervention to “yes” two weeks after the intervention, indicating an increase in children’s positive evaluations of smartphone-based education. |
| Kang et al [34]  2017 | South Korea | Descriptive study (app development and usability testing) | Teachers | 89 teachers | App (S-IPA) | In-house development; not publicly available | ①⑥ | — | — | ^r^US | Overall satisfaction average score: 7.7 ± 1.14 (out of 10) |
| Schwebel et al [30]  2018 | China | Quasi-experimental study | Children aged 8-10 | 56 | Smartphone-based virtual reality (VR) + wearable devices (VR goggles) | In-house development, created by the research team in 2017, is not publicly available but still under development | ⑧ | — | 12 days | ^j^Behavior  ^t^UEx  ^p^Self-Efficacy | Children’s traffic safety behaviors improved significantly after training. Self-efficacy increased from 3.6% to 19.6% (OR=4.7), and more than half (57.1%) of the students found the VR training interesting and meaningful. |
| Burgess et al [27]  2018 | Australia | RCT | Parents of children aged 5-12 months | 262/236 | App (Cool Runnings) | Developed by a research team at the University of Queensland’s Center for Pediatric Burns and Trauma, but it is currently unavailable for download. | ①⑥⑧ | Used the same app, but without gamification strategies | 6 months | ^e^Knowledge  ^s^UE | This study significantly improved mothers’ knowledge of the risks of hot-beverage scalds and of burn first aid. The proportion of mothers with adequate knowledge in the intervention group was nearly three times that in the control group. Gamification techniques were significantly associated with improved knowledge (*P*<.01). However, the dropout rate was high (51%), and only 27.3% of the intervention group was classified as “high engagement.” |
| Ning et al [25]  2019 | China | RCT | Caregivers of preschool children aged 3-6 | 1,073/907 | App (Bao Hu San) | In-house development; not publicly available | ①②③④ | Same app, but it does not provide accident prevention education. | 6 months | ^a^Incidence  ^f^Attitude  ^h^Behavior  ^x^EL  ^y^EC | During the 6-month follow-up period, there were no significant changes in the incidence of unintentional injuries or attitude scores in either group. Improvements in safety behaviors were superior to the control group ($\beta$=0.87, 95% CI 0.33-1.42, *P*=.002). Additionally, due to difficulties with data collection and the absence of significant differences in primary outcomes, analysis of economic losses resulting from unintentional injuries and the app’s economic costs were not conducted. |
| Roberts et al [32]  2019 | United States | Mixed methods study | Children aged 0-12  Parents of children | — | App (Make Safe Happen) | Developed by experts at the US Center for Child Injury Prevention. This app is designed to help parents and caregivers of children aged 0-12 prevent household accidents (such as poisoning, falls, burns, and drowning). The app is currently unavailable for download. | ①⑥⑦ | — | 7-10 days | ^h^Behavior  ^q^CAIP  ^z^BFF  ^s^UE  ^t^UEx | Prevention awareness increased significantly from 63% before the test to 81% (*P*<.01). The completion rate of home safety behaviors increased by 45%. Participants rated their own home safety an average of 2.7 points (range 2.5-2.9), slightly higher than the safety standard for most households; Parents (73%, 29/40) reported a positive experience using the app. During the usage period, parents opened the app 229 times, viewed 3,314 screens, performed 1,972 safety actions, and added safety products to their shopping lists 19 times. 87% of parents intended to take safety measures in the future, but lack of time, poor relevance of information, product cost, and installation difficulties were the main barriers. |
| Dixon et al [23]  2019 | US | RCT | Children aged 5-6 | 30/30 | Game app (iBsafe) | In-house development; not publicly available | ①②⑧ | American Academy of Pediatrics Safety Guide | 1 week | ^c^Knowledge  ^h^Behavior  ^s^UE  ^u^UA | After the intervention, children significantly outperformed the control group in both bicycle and dog safety knowledge and skills. The proportion of parents who rarely discussed dog safety dropped from 83% before the intervention to 33% (compared to only 67% in the control group). In addition, 87% of children enjoyed the game, and 83% of parents rated it as “good/very good.” Nearly all parents acknowledged its educational effectiveness and expressed a willingness to purchase it. |
| Yan et al [26]  2020 | China | RCT | Preschool children Parents | 69.74/63 | WeChat | WeChat group | ①②⑧ | Traffic safety training (excluding CRS) | 3 months | ^d^Knowledge  ^g^Attitude  ^k^Behavior  ^v^CRSR  ^r^US | CRS usage in the intervention group was significantly higher than in the control group (OR 0.29, 95% CI 0.11-0.79, *P*=.016), 8 out of 9 indicators of knowledge, attitude, and behavior showed significant improvement. Over 90% of parents endorsed the intervention measures in this study. |
| Jones et al [33]  2020 | United Kingdom | Qualitative study (app development and usability testing) | Caregivers of children under 5 | 15 mothers | App (Grow up Safely) | In-house development; not publicly available | ①⑦ | — | — | ^s^UE  ^t^UEx | Participants approved of the app’s name and layout. Most parents found the health information comprehensive and the images intuitive, but young mothers reported that the language was verbose and not sufficiently intuitive enough, and that a few images were not closely related to the topic. Most participants indicated they would be willing to download and use the app following a professional recommendation. Most parents recognized the app’s concept and usefulness, but the group of young mothers felt the content leaned toward common knowledge and were unwilling to read it proactively. Suggestions included adding information on first aid and childhood illnesses, and implementing regular push notifications. |
| Choi et al [22]  2021 | South Korea | RCT | Primary caregivers of infants and toddlers | 59, 53/54 | Mobile-based e-learning program | Developed using Storyline 360 e-learning software, the program generates a course link that includes videos, audio, and slides. Parents can access the link via computers, tablets, and smartphones and learn at their own pace; the course supports repeated viewing and progress tracking. This program is not available to the public. | ① | Blank | 2 weeks | ^b^Knowledge  ^h^Behavior  ^r^US | The intervention group significantly outperformed the control group in safety behaviors (*F=*10.09, *P*<.001) whereas there were no significant differences in safety knowledge or satisfaction with the instruction. |
| McKenzie et al [24]  2021 | US | RCT | Children aged 0-12 Parents | 4,182/850 | App (Make Safe Happen) | Developed by experts at the US Center for Child Injury Prevention, this app is designed to help parents and caregivers of children aged 0-12 prevent household accidents (such as poisoning, falls, burns, and drowning). The app is currently unavailable for download. | ①⑥⑦ | Use other apps | 1 week | ^c^Knowledge  ^h^Behavior | After the intervention, the intervention group demonstrated superior safety knowledge, one-time safety behaviors, and repeated safety behaviors compared with pre-intervention levels and with the control group (*P*<.0001). |
| Feng et al [15]  2022 | China | RCT | Parents of children aged 0-3 years | 182/183 | WeChat | The research team established a WeChat group and registered a WeChat official account (Children’s Safety and Health). The account is currently searchable, but is no longer being updated. | ①② | Join the WeChat group only | 12 months | ^a^Incidence  ^e^Knowledge  ^i^Behavior  ^n^Beliefs  ^y^EC | Following the intervention, the incidence of unintentional injuries among children in the intervention group was lower than in the control group (OR*=*1.71, 95% CI 1.02-2.87, *P*=.04). Knowledge showed no significant difference (*P*=.06). Improvements in beliefs and behaviors were significantly greater in the intervention group than in the control group (*P*<.001). The cost-effectiveness of the intervention was 4.83 to 5.26 times the cost. |
| Wong et al [17]  2024 | Hong Kong, China | RCT | Children aged 8-13 | 170/170 | Mobile smartphone-based serious game app (Safe City) | Developed by the research team in 2020, available for download via a web browser, but requires a mobile phone number to register | ①⑧ | Safety manual education | 30 days | ^c^Knowledge  ^h^Behavior  ^o^Psychosocial | There were no significant differences between the intervention and the control groups in the mean changes in safety knowledge, safety behaviors, internalizing problems, or externalizing problems (all *P* > 0.05). However, the dose-response relationship indicated that cumulative game scores were associated with improvements in safety behavior (*P*=.03) and internalizing problems (*P*=.01) at the 3-month mark of the intervention. Performance on the Matching Game and the Spot the Danger mini-game significantly predicted improvements in safety knowledge at 1 month and 3 months post-intervention. |
| Cooray et al [16]  2024 | Australia | Mixed methods study | Parents of infants aged 0-1 years (within 2 months of the due date) | 60/10 (interviews) | App | In-house development; not publicly available | ①②⑥ | — | 3 months | ^l^Behavior  ^z^BFF  ^s^UE  ^u^UA | Following the intervention, the average total score for parents’ overall potential to reduce fall risk increased from 15.77 ± 2.24 to 18.05 ± 1.86 (*P*<.001). The overall user acceptance score was 4.05 ± 0.87 (out of 5), indicating above-average user acceptance within an acceptable range, though room for improvement remains. Qualitative data revealed that user acceptance and engagement were limited by impractical recommendations (e.g., reliance on social support, the inapplicability to mothers with multiple children or those who had undergone cesarean sections), difficulty in using the task tracker, and content that focused solely on falls while lacking coverage of first aid and a broader age range. |
| He et al [28]  2025 | China | RCT | Caregivers of rural preschool children aged 3-6 | 1,662/1,676 | App (Bao Hu San) | He et al. adapted the content to rural contexts, increasing the proportion of audio-visual materials and interactive games while reducing text and images, and added online forums and themed activities to strengthen interaction between researchers and caregivers. | ①②③④ | Conventional School Education | 18 months | ^a^Incidence  ^f^Attitude  ^i^Behavior  ^m^Environment | The 12-month incidence of unintentional injuries in the intervention group was significantly lower than in the control group (aRR 0.93, 95% CI 0.86-0.98). The incidence of falls was slightly higher than in the control group (aRR=1.17, 95% CI 1.06-1.30). Attitudes, behaviors, and home safety were significantly better than in the control group. |
| Müller et al [31]  2025 | Germany | Mixed methods study (usability evaluation of an existing app) | Childcare providers | 21 parents of children under 7 years of age  21 temporary caregivers | App (Vergiftungsunfalle bei Kindern) | Developed by the German Federal Institute for Risk Assessment in 2013, available for download on Google Play and the App Store. | ①⑦ | — | — | ^t^UEx  ^z^BFF_1_ | Users praised the app’s clear structure, practical alphabetical index, and search functions, and the content that helps identify underestimated dangers. However, the text lacks coherence, uses too much specialized language, and features inconsistent information, leading to difficulties in understanding and time-consuming use; some users felt the information was too basic or unnecessary, and suggested adding plant photo identification, visual hazard ratings (e.g., traffic light system), faster emergency call functions, and links between modules. |

① Health education; ② Interactive communication; ③ Surveys and documentation; ④ Personalized settings; ⑤ Evaluation; ⑥ Monitoring and reminders; ⑦ Emergency settings; ⑧ Behavioral training.

^a^Incidence: Incidence of childhood accidents.

^b^Knowledge: awareness of unintentional injuries.

^c^Knowledge: knowledge of prevention and safety.

^d^Knowledge: knowledge of child restraint system use.

^e^Knowledge: first aid knowledge in the event of an accident.

^f^Attitude: caregivers’ attitudes toward accident prevention.

^g^Attitude: the use of child restraint systems.

^h^Behavior: injury prevention and safety behaviors.

^i^Behavior: caregiver supervision behaviors.

^j^Behavior: traffic safety behaviors.

^k^Behavior: the use of child restraint systems.

^l^Behavior: behaviors with the potential to reduce the risk of infant falls.

^m^Environment: home environment safety;

^n^Beliefs: caregivers’ beliefs regarding the preventability of unintentional injuries;

^o^Psychosocial: psychosocial problems in children;

^p^Self-Efficacy: children’s self-efficacy when crossing the street;

^q^CAIP: caregivers’ awareness of injury prevention;

^r^US: User Satisfaction;

^s^UE: User Engagement;

^t^UEx: User Experience;

^u^UA: User Acceptance;

^v^CRSR: the utilization rate of child restraint systems;

^w^EES: The educational effectiveness of smartphones (frequency, practicality, interest, and ease of understanding);

^x^EL: Economic losses resulting from childhood accidents;

^y^EC: The economic costs of the apps;

^z^BFF: the barriers and facilitators to caregivers’ adoption of unintentional injury prevention measures;

^z^BFF_1_: the barriers and facilitators affecting user acceptance.
